# Puerperal Eclampsia*Graduating Thesis of R. T. Morris, M. D., Houston, Texas, late resident student Charity Hospital, N. O.: One of three selected for publication by the Faculty of the Tulane University Medical College, out of the one hundred graduating theses presented.

**Published:** 1894-11

**Authors:** Robert T. Morris

**Affiliations:** Houston, Texas


For Texas Medical Journal.

### PUERPERAL ECLAMPSIA.*

BY ROBT. T. MORRIS, M. D., HOUSTON, TEXAS.

THE mortality of this disease, maternal and infantile, and the antagonistic views of various authorities, as to its ætiology and treatment, are sufficient excuses for the presentation of these cases and the deductions made therefrom.

Writers differ also in regard to its frequency: Cazeaux writes that it occurs once in every two hundred cases of labor; Parvin places the ratio at 1 to 300; Meigs, 1 to 500. Most authors agree with the last estimate. In examining some of the records of the Charity Hospital of New Orleans, I noticed that there were nineteen cases of eclampsia in 1552 deliveries, or 1 to 81. This discrepancy is due to the fact that a great number of cases were brought to the hospital because they were eclamptics, and if pregnancy had been uncomplicated, they would have received attention elsewhere; furthermore, their sanitary surroundings, previous to entrance, were none of the best, and, as a rule, no prophylaxis was observed.

The ætiology of this disase is, as far as I can ascertain, clouded in mystery. I believe that he who contends that one factor is the causative agent, and overlooks the several conditions operating in the production of this disease, will see many cases not reconcilable to his theorem. While fully seven-tenths of the

---

* Graduating Thesis of R. T. Morris, M. D., Houston, Texas, late resident student Charity Hospital, N. O.: One of three selected for publication by the Faculty of the Tulane University Medical College, out of the one hundred graduating theses presented.

cases are of nephritic origin, we must not forget the remaining, which may be accounted for by the Traube-Rosenstein theory, or may be due to uterine irritation. The pressure and irritation of the child *in utero* has undoubtedly a causative influence, or one-third of the cases of convulsions would not cease after delivery, spontaneous or otherwise. (Pavrin.) If we consider the hydraæmic condition of the blood of the pregnant woman; the retention of excrementitious matter in the system, whether it be urea, acetone, carbonate of ammonia, or ptomains; and in connection remember the convulsibility of the "centers," and the undoubted effect of uterine irritation, we will, in the great majority of cases, be able to account satisfactorily for the phenomena presented.

Admitting the nephritic origin of the disease, we are still at a loss to account for the convulsions. Why should excrementitious matter circulating in the blood produce clonic convulsions, insensibility, sterterous respiration, and the train of symptoms so often observed? King presents a very ingenious theory for the production of the disease. He contends that the normal position of the fœtus during pregnancy is oblique, and any deviation from this position produces pressure upon the abdominal vessels, which results in renal trouble and cerebral hyperæmia. His treatment is a logical sequence; remedy the malposition and you will prevent the convulsions.

PROGNOSIS.—Madam La Chapelle estimates the maternal mortality at 50 per cent.; Cazeaux, 33 per cent., and Braun, 26 per cent. The infantile mortality is about 50 per cent. The prognosis is influenced by the time of the occurrence of the convulsions, the earlier they occur in pregnancy the more unfavorable the result, and when they occur first after delivery, the more favorable is the result. In one of the cases reported the convulsions occurred seven days after the completion of labor. Boilly saw a case twenty-nine days after labor; Simpson mentions a case eleven days after delivery.

The following cases are not as complete as I wished, but I hope their accuracy will partially compensate for their brevity:

CASE 1.—M. B., 16, primipara. Nine hours after delivery, patient had a single convulsion, another some hours later. Bromide and chloral were given every hour and a half. Discharged well; child, well.

CASE 2.—M. R., 19, primipara. Convulsions fifteen minutes after delivery, controlled by chloroform, bromide and chloral.

Albumin found in urine fifteen days before confinement. Discharged well; child, well.

CASE 3.—M. W., primipara. Twelve or fourteen convulsions during labor, but ceased after delivery. Urine contained 20 per cent. albumin. Chloroform, bromide and chloral. Died seven days after delivery from septecæmia; child stillborn.

CASE 4.—S. B., 25, multipara. Convulsions during and after labor. Patient delivered with forceps. Bromide and chloral. Died; child, stillborn.

CASE 5.—S. F., primipara. Several convulsions before delivery, but none after. Bromide and chloral. Died six days later from septicæmia; child, well.

CASE 6.—J. N., 35, multipara. Convulsions after delivery. Bromide, chloral and chloroform. Discharged well; child died with convulsions.

CASE 7.—M. B., 21, primipara. Convulsions during labor, but none after delivery. Bromide, chloroform and cathartics. Discharged well; child, stillborn.

CASE 8.—M. L., 22. Convulsions after delivery; usual treatment. Died. An autopsy showed a fatty and ecchymotic kidney.

CASE 9.—M. A., 24, primipara. Convulsions before and after delivery. Delivered with forceps. Patient had a mitral murmur. Chloral, bromide and chloroform. Died. Autopsy disclosed fatty kidneys and liver. Child, stillborn.

CASE 10.—M. C., 18, primipara. Convulsions during and after labor; forceps; usual treatment. Died; child, stillborn.

CASE 11.—F. W., 22, primipara. Convulsions after delivery; forceps; usual treatment. Died eight weeks later from empyema; child, stillborn.

CASE 12.—M. R., 19, primipara Patient six months pregnant. Convulsions ceased after delivery. Pilocarpine, morphine, and hot cans. Urine contained 60 per cent. of albumin. Discharged well; child, stillborn.

CASE 13.—M. R., 19, primipara. Convulsions three hours after delivery and reached seven in number. Pilocarpine, bromide, chloral and chloroform. Urine contained albumin. Discharged well; child, well.

CASE 14.—L. J., 30, primipara. Convulsions during and after delivery. Patient anasarcous. Urine contained 10 per cent. of albumin. Morphine, bromide, chloral and chloroform. Discharged well; child, stillborn.

CASE 15.—L. S., 16, primipara. Convulsions during labor, but none after; forceps. Urine, 15 per cent. of albumin, granular and hyaline casts. Discharged well; child, stillborn.

CASE 16.—E. P., 17, primipara. Convulsions during and after delivery; forceps. Discharged well; child, well.

CASE 17.—M. S., 21, multipara. Convulsions before admission. Temperature, 105° at time of entrance; forceps, antifebrine and digitalis. Died; child, stillborn.

CASE 18.—M. G., 21, primipara. Eight months pregnant. Previous history of convulsions. There were over thirty convulsions, and patient remained in a semi-comatose condidion for forty-eight hours. Urine contained albumin, granular and hyaline casts. Pilocarpine, chloral and bromide. Discharged well; child, stillborn.

CASE 19.—M. G., 16, primipara. Four convulsions seven days after delivery. Pilocarpine, morphine, bromide and chloral. Discharged well; child, well.

In addition to the above cases it may be of interest to report five cases of pregnancy which presented every evidence of organic changes in the kidneys, but at no time did they show symptoms of impending eclampsia.

CASE 1.—A. S. Urine contained 38 per cent of albumin, granular and hyaline casts. Discharged well; child stillborn.

CASE 2.—M. F., 38., primipara. 60 per cent. of albumin, granular and hyaline casts. Died a few days after delivery; child stillborn.

CASE 3.—R. S., 24, primipara; 25 per cent. of albumin, granular and hyaline casts. Discharged well; child well.

CASE 4.—V. T., 29, multipara. General anasarca, granular and hyaline casts. Discharged well; child well.

CASE 5.—S. W., 19, primipara. 20 per cent. of albumen, granular and hyaline casts. Died from suppression of urine; child well.

It will be observed that fifteen of the nineteen cases occurred in primipara; that in seven of the cases the convulsions occurred previous to labor, with a mortality maternal of 14 per cent., and infantile of 85 per cent.; that in six of the cases the convulsions occurred during and continued after delivery; mother, 50 per cent; child, 80 per cent.; that the remaining six cases were subsequent to delivery, with a maternal mortality of 16⅔ per cent; infantile, $33\frac{10}{38}$. One of the children died with convulsions. The following is the total mortality: mother, 26 per cent.; child, 62

per cent. It is a noteworthy fact that 36.8 per cent. of the cases ceased after the emptying of the uterus.

TREATMENT.—The management of these cases is divided into prophylactic and curative. Beyond question successful prophylaxis is the ideal treatment, but we are not always so fortunate as to ward off the disease, but the danger can be minimized by a careful and persistent examination of the urine to ascertain the presence of albumen or a diminution in the excretion of urea, or the formation of granular or hyaline casts. The efficacy of a regulated diet, hygiene, and an even and unexcited mode of living should be remembered.

The curative treatment may be considered under four headings, viz.: obstetrical management, sedatives, eliminants, and bleeding.

1. The obstetrical management, when applicable, should take precedence of all therapeutic measures. When 33 per cent. of the cases cease, and 33⅓ of the remaining diminish in intensity after obstetrical interference, its efficacy becomes obvious and too pronouced to be disregarded, notwithstanding the edict of Gooch, "attend to the convulsions and leave the labor to take care of itself."

2. The sedatives in most general use are chloroform, bromide, chloral, morphine, and veratrum viride. Very encouraging results have been reported from the intermittent use of chloroform, but in a good many cases morphine, hypodermatically, will diminish the intensity and prolong the interval between the convulsions, and at the same time diminish renal congestion and assist the diaphoretic action of pilocarpine, which can be given after the administration of the morphine. Veratrum viride has some extremely enthusiastic advocates.

3. There are many eliminants, but pilocarpine, in my opinion, is the best. It may be given in ⅙ grain doses, every two hours, until free diaphoresis ensues. Murphy recommends ⅓ of a grain every six hours, but a better effect is obtained by smaller doses more frequently repeated. To obviate bronchorrhœa and increase the action of the pilocarpine, hot baths and drinks should be used. Many observers affirm that pilocarpine stimulates the gravid uterus; if this is true, the drug possesses another virtue in the treatment of the disease. It has been reported that the skin eliminates from fifteen to seventeen grains more of urea under the use of pilocarpine, but while an increased amount of urea in the system is inert, it is reasonable to suppose that toxic excrementitious matter is eliminated with it, and the good effect is then obtained. Elaterium is one of the best of the many cathartics. One great advantage is that the condition of unconsciousness is no contra-indication to its use.

4. The history of bleeding in eclampsia illustrates the various cycles a measure may pass through before it reaches its therapeutic level. A few years ago bleeding was considered specific in this disease, and every obstetrician carried his lancet, but at the present time bleeding is seldom resorted to.

Listen to the words of Cazeaux: "As a curative measure we must place sanguineous emission at the head of the list." Meigs cries out in defense of this measure: "The lancet, the lancet, and nothing but the lancet is worthy of confidence."

Delivery, pilocarpine, morphine, and cathartics appeal to me as the most rational agents at our disposal.

